# Auranofin displays anticancer activity against ovarian cancer cells through FOXO3 activation independent of p53

**DOI:** 10.3892/ijo.2014.2579

**Published:** 2014-08-04

**Authors:** SEE-HYOUN G PARK, JUN GHAN LEE, JONATHAN S. BEREK, MICKE Y C.-T. HU

**Affiliations:** 1 Division of Gynecologic Oncology, Department of Obstetrics and Gynecology, Stanford University School of Medicine, Stanford, CA 94305, USA; 2 Department of Obstetrics and Gynecology, Hanyang University School of Medicine, Seoul 133-792, Republic of Korea

**Keywords:** auranofin, cancer, FOXO, Forkhead, apoptosis, caspase, Bax, Bim, p53, IκB kinase, tumor suppression

## Abstract

Auranofin is a gold-containing compound classified by the World Health Organization as a clinically established rheumatoid arthritis therapeutic agent. Through drug screening for novel anticancer therapeutics, we unexpectedly identified auranofin as a potent anticancer agent against a p53-null ovarian carcinoma SKOV3 cell line. However, the molecular mechanism underlying auranofin-mediated anticancer activity in ovarian cancer cells is basically unknown. Here, we show that auranofin inhibits proliferation and survival of SKOV3 cells in a dose- and time-dependent manner. Auranofin treatment activates the pro-apoptotic caspase-3, increases protein levels of apoptosis-inducing proteins Bax and Bim and reduces the expression of the anti-apoptotic mediator Bcl-2 in SKOV3 cells. Moreover, auranofin downregulates IκB kinase (IKK)-β and promotes nuclear localization and the activation of FOXO3 tumor suppressor, leading to cellular apoptosis in SKOV3 cells. In contrast, silencing FOXO3 diminishes the pro-apoptotic signaling of auranofin in SKOV3 cells. These results suggest that auranofin may induce caspase-3-mediated apoptosis in a FOXO3-dependent manner. The observed upregulation of pro-apoptotic genes and apoptosis in cancer cells without p53 in response to auranofin suggests a novel p53-independent mechanism underlying auranofin-induced apoptosis in ovarian cancer cells.

## Introduction

Ovarian cancer is the leading cause of gynecologic cancer deaths in the United States (US) (1). While ovarian cancer has a typically good response to first-line combination chemotherapy after initial cytoreductive surgery, the prognosis of patients with advanced malignant ovarian cancer remains poor because of acquired chemotherapy resistance (2). Despite recent advances made in chemotherapies of ovarian cancer, the overall survival of patients has not improved significantly because a considerable number of patients harbor ovarian cancer refractory to these therapies and the majority of the initially responsive tumors become resistant to treatments (3). Thus, the development of novel targeted therapy that retains activity against chemotherapy-resistant ovarian cancer is an unmet and urgent medical need.

**Figure 1:**
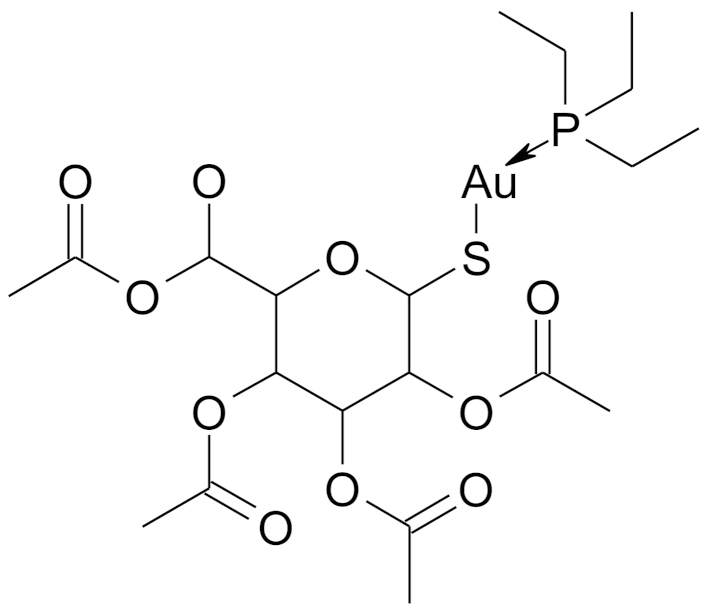
Chemical structure of auranofin.

Auranofin [2,3,4,6-tetra-o-acetyl-1-thio-β-D-glucopyranosato-S-(triethyl-phosphine) gold] is a thiol-reactive gold (I)-containing compound (Fig. 1) that reduces the effects of the inflammatory process in the body, and has been utilized to treat rheumatoid arthritis by reducing pain and swelling (4,5). Auranofin can inhibit IgE- and non-IgE-mediated histamine release from human basophils and suppress the *de novo* synthesis of sulfidopeptide leukotriene C4 (LTC4), which is induced by anti-IgE from basophils and mast cells (6). In addition, auranofin is a potent inhibitor of mitochondrial thioredoxin reductase *in vitro* and *in vivo*, since auranofin is able to block its active site (7). In addition, auranofin has been shown to inhibit the activation of the IKK/NF-κB signaling pathway and thereby downregulate the gene expression of pro-inflammatory cytokines such as IL-1β and TNF-α (8–10). Our previous study shows that IKK-β activation phosphorylates the serine-644 residue in the FOXO3 protein and subsequently triggers faster protein degradation of FOXO3 via the proteasome-mediated ubiquitination (Ub) mechanism, which results in the stimulation of human breast cancer cell proliferation *in vitro* and the development of breast tumor *in vivo* (11,12). These findings suggest that IKK-β may be a potential target for anticancer therapy through FOXO3 activation. Interestingly, when we screened for small molecules that can promote the activation of FOXO3 in ovarian cancer cells, we identified auranofin as a candidate FOXO3-activating small molecule from the US Food and Drug Administration (FDA)-approved compound libraries. Since auranofin is also a candidate inhibitor targeting IKK-β, it is a promising small molecule candidate for development as anticancer therapeutics.

FOXO3 is a member of the human Forkhead-box (FOX) gene family that is known to have the distinct Forkhead DNA binding domain (13). As a transcriptional factor, FOXO3 is known to regulate various cellular processes such as cell cycle (14,15), cellular apoptosis (16–19), DNA damage repair (20,21), stress responses (15,22,23), metabolism (24), aging (25) and tumor suppression in mammalian cells (12,18,26). The results from gene knockout exhibit key functions of FOXO family members in tumor suppression (27) and in preventing the decline of the hematopoietic stem cell pool (28). The FOXO3 protein in cancer cells can be regulated by various protein modification mechanisms such as phosphorylation, acetylation and ubiquitination (25,29–31). It has been shown that several active protein kinases (such as Akt, IKK-β and MAPK) display the ability to phosphorylate the specific serine/threonine residues on the FOXO3 protein and induce the translocation of FOXO3 from the nucleus to the cytoplasm. This nuclear exclusion and translocation of FOXO3 into the cytoplasm inhibits FOXO3-dependent transcription and results in the proteasome-mediated Ub and protein degradation of FOXO3 (11,12,18,25,31). This inhibition of function of FOXO3 leads to tumor development and progression, suggesting that FOXO3 is a crucial tumor suppressor. Importantly, several clinical studies on the relationship between the FOXO3 protein nuclear localization or expression level and cancer patient survival rates have revealed that FOXO3 is a good prognostic biomarker for cancer survival (12,32–34), which suggests that the regulation of FOXO3 activation in cancer cells may be a promising strategy for developing anticancer therapeutic drugs.

Sequential activation of caspases (the cysteinyl aspartate-specific proteases) plays an important role in the execution phase of cellular apoptosis (35). In general, caspases exist as inactive pro-enzymes that undergo proteolytic processing at the conserved aspartic residues to produce two fragments that form a functional dimer as an active protease. It has been shown that cleavage of poly(ADP-ribose) polymerase 1 (PARP1) by caspase-3, a crucial protease in regulating apoptosis, at the pro-apoptotic cleavage site of PARP1 produces the p85-kDa proteolytic fragment, which has been suggested as a biomarker associated with apoptosis (36,37).

In the present study, we used human ovarian carcinoma SKOV3 cells, which are p53-null, as the cell model system to investigate the cytotoxic activity and the anticancer mechanisms of auranofin. Cell-based assays and biochemical analyses were employed to elucidate the molecular mechanism underlying the anticancer activity of auranofin in SKOV3 cells. Based on our findings, we propose that FOXO family members may upregulate the pro-apoptotic genes and downregulate certain cell survival genes that contribute to the cellular apoptosis response to auranofin in a p53-independent manner. The important biological and pathological significance of this new mechanism of auranofin in cancer therapy is discussed below.

## Materials and methods

### Chemical reagents and antibodies

Auranofin, dimethylsulfoxide (DMSO), glycerol, glycine, sodium chloride, thiazolyl blue tetrazolium bromide, Trizma base and Tween-20 were purchased from Sigma (St. Louis, MO, USA). Mouse anti-IκB kinase β (1:2,000 dilution), mouse anti-IκBα (1:1,000 dilution), rabbit anti-p-IκBα (1:1,000 dilution), mouse anti-PARP1 (1:1,000 dilution) and rabbit anti-FOXO3 (1:1,000 dilution) antibodies were obtained from Santa Cruz Biotechnology (Santa Cruz, CA, USA). Rabbit anti-cleaved caspase-3 (1:1,000 dilution), rabbit anti-Bax (1:1,000 dilution), rabbit anti-Bim (1:1,000 dilution) and rabbit anti-Bcl-2 (1:1,000 dilution) antibodies were purchased from Cell Signaling Technology (Danvers, MA, USA). Mouse anti-β-actin antibody (1:3,000 dilution) was purchased from Sigma. Goat anti-mouse and goat anti-rabbit horseradish peroxidase-conjugated IgG were obtained from Jackson ImmunoResearch (West Grove, PA, USA). ECL Western Blotting Detection reagents were obtained from Genedepot (Barker, TX, USA).

### Cells, cell culture and siRNA transfection

Human ovarian carcinoma SKOV3 cells (from ATCC) were maintained in DMEM/F12 media supplemented with 10% fetal bovine serum, 3% L-glutamine and 1% streptomycin/penicillin at 37°C in a humidified incubator containing 5% CO_2_ in air. FOXO3-siRNA and control-siRNA were obtained from Santa Cruz Biotechnology. Cells were transfected with FOXO3-siRNA or control-siRNA by using DharmaFECT 1 transfection reagent (Thermo Scientific, Rockford, IL, USA), according to the manufacturer’s instructions and as described previously (19).

### MTT cell viability assay

A 200 μl aliquot of cells (1×10^3^ cells in media) was added to each well of a 96-well plate and incubated for 18 h at 37°C in a humidified incubator containing 5% CO_2_ in air. After incubation, each dose (0, 50, 100, 200 and 400 nM) of auranofin was added into the wells for 72 h for the dose-dependent response assay and 100 nM of auranofin was added into the wells for 0, 24, 72 and 120 h for the time-dependent response assay. Control cultures were treated with DMSO. After incubation, a 20 μl MTT solution (5 mg/ml in phosphate buffer) was added to each well and the incubation continued for 4 h, after which time the solution was carefully removed. The blue crystalline precipitate was dissolved in DMSO 200 μl. The visible absorbance at 560 nm of each well was quantified using a microplate reader.

### Cell counting assay

SKOV3 cells (1×10^4^) were seeded in 6-cm dishes and incubated at 37°C in a humidified incubator containing 5% CO_2_ in air incubator for 18 h. After incubation, cells were treated with DMSO as control vehicle and the indicated concentration of auranofin (100 nM) for 0, 24, 72 and 120 h. Each day, cell numbers were measured by using a hemocytometer.

### Colony formation assay

SKOV3 cells (0.5×10^3^) were seeded in 6-cm dishes and incubated at 37°C in a humidified incubator containing 5% CO_2_ in air incubator for 18 h. After incubation, cells were treated with DMSO as control vehicle and the indicated concentration of auranofin (100 nM) for 7 days. The colonies were washed twice with PBS, fixed with 3.7% paraformaldehyde and stained with 1% crystal violet solution in distilled water.

### Western blot analysis

Western blot analysis was performed as described previously (11,12,19). Briefly, cells were washed with PBS and lysed in lysis buffer (50 mM Tris-HCl, 150 mM NaCl, 2 mM EDTA, 1% Triton X-100, 0.1% SDS, pH 8.0) with protease and phosphatase inhibitors. Cell lysates were centrifuged (10,000 × g, 4°C, 10 min) and the supernatants were separated on 6 or 10% SDS-PAGE gels and blotted onto nitrocellulose membranes (Bio-Rad Laboratories, Hercules, CA, USA). The membranes were blocked in 3% non-fat dry milk for 1 h at room temperature and probed with appropriate antibodies. Membranes were then probed with HRP-tagged anti-mouse or anti-rabbit IgG antibodies diluted 1:5,000–1:15,000 in 3% non-fat dry milk for 1 h at room temperature. Chemiluminescence was detected using enhanced ECL.

### Cytoplasmic and nuclear protein fractionation

Cells from each condition were trypsinized, centrifuged, washed, re-suspended in a cytoplasmic fractional buffer (10 mM HEPES, pH 8.0, 50 mM NaCl, 500 mM sucrose, 1 mM EDTA, 0.5 mM spermidine, 0.15 mM spermine, 0.2% Triton X-100, 1 mM DTT, 2 μM PMSF and 0.15 U/ml aprotinin) and incubated at 4°C for 30 min on a rotator. The cell suspension was centrifuged at 10,000 rpm for 30 min at 4°C and the supernatant was collected for cytoplasmic fraction. The nuclear pellet was washed twice with the washing buffer (10 mM HEPES pH 8.0, 50 mM NaCl, 25% glycerol, 0.1 mM EDTA, 0.5 mM spermidine and 0.15 mM spermine). The remaining pellet was re-suspended with a nuclear fractional buffer (10 mM HEPES pH 8, 350 mM NaCl, 25% glycerol, 0.1 mM EDTA, 0.5 mM spermidine and 0.15 mM spermine) and incubated at 4°C for 30 min on a rotator. The nuclear suspension was centrifuged at 13,000 rpm for 30 min at 4°C, the supernatant was collected for nuclear fraction. Protein in each fraction was quantified by the Bradford protein determination reagent (Bio-Rad Laboratories), using BSA as a standard.

### DNA fragmentation assay

SKOV3 cells (2×10^7^ per sample) were trypsinized, lysed in the lysis buffer (10 mM Tris-HCl, 10 mM EDTA, 0.1% Triton-X 100, 0.1% SDS and pH 7.5) and incubated on ice for 30 min. The lysates were digested with RNase I followed by digestion with proteinase K. The DNA was extracted by phenol-chloroform (1:1, v/v), precipitated with 2 volumes of EtOH plus 10% NaAc (3 M, pH 5.2) and then dissolved in distilled water. Equal amounts of the extracted DNA (2 μg/lane) and size markers (1-kb ladder) were subjected to electrophoresis on 2% agarose gels, which were stained with ethidium bromide and photographed.

### Statistical analysis

Results are expressed as arithmetic mean ± SEM (the standard error of the mean). To compare the statistical meaning between the groups, two-sided unpaired Student’s t-test was used. All experiments were repeated three times and the representative data are shown. Statistical analyses were performed using SPSS software (version 19.0, SPSS Inc., Chicago, IL, USA). Mean differences with P-values <0.05 were considered statistically significant.

## Results

### Auranofin inhibits cell survival or growth of SKOV3 cells

**Figure 2:**
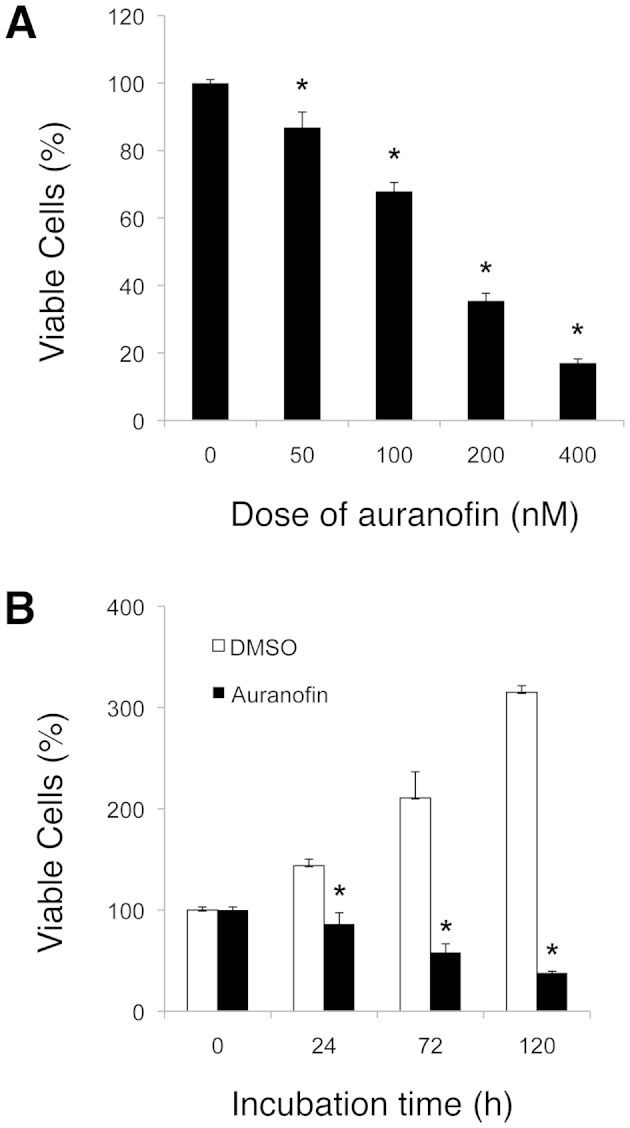
The cytotoxic effect of auranofin on SKOV3 cells. (A) The dose-dependent effect of auranofin (0, 50, 100, 200 and 400 nM) on SKOV3 cells after 72-h incubation. (B) The time-dependent effect of auranofin (100 nM) on SKOV3 cells after 0, 24, 72 and 120 h. The cell viability was determined by the MTT assay and the relative cell survival rate percentage was calculated by dividing the optical density of each auranofin treatment by the optical density of the control (DMSO) treatment. The significant P-values between the auranofin group versus the control group are indicated (^*^P<0.001). The results are based on three replicates. The error bars represent standard deviation by paired t-test.

**Figure 3:**
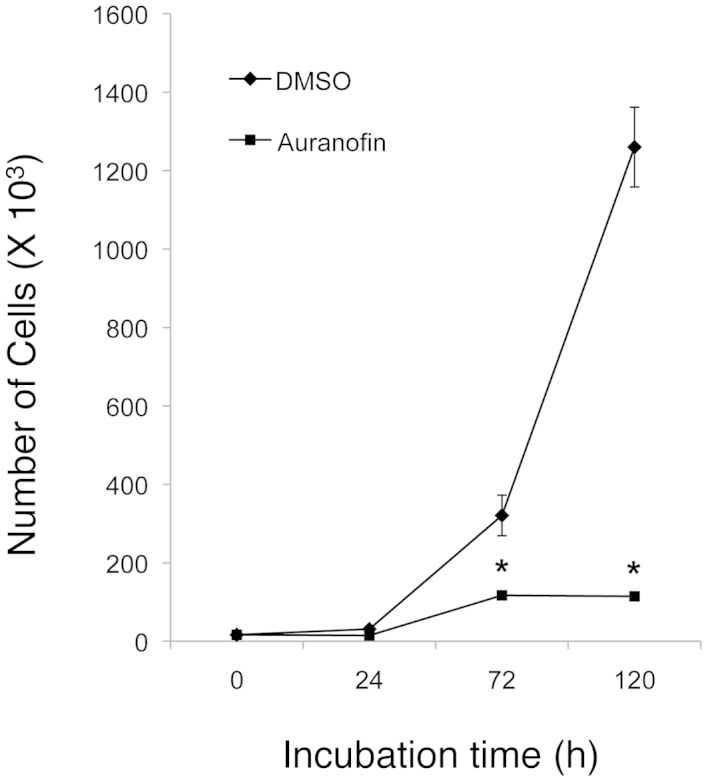
Auranofin inhibits cell survival or growth of SKOV3 cells. The cell numbers were determined by the cell counting assay after SKOV3 cells (1×10^4^ cells/plate) were treated with auranofin (100 nM) or the control (DMSO) for 0, 24, 72 and 120 h. The significant P-values between the auranofin group versus the control group are indicated (^*^P<0.001). The results are based on three replicates. The error bars represent standard deviation by paired t-test.

**Figure 4:**
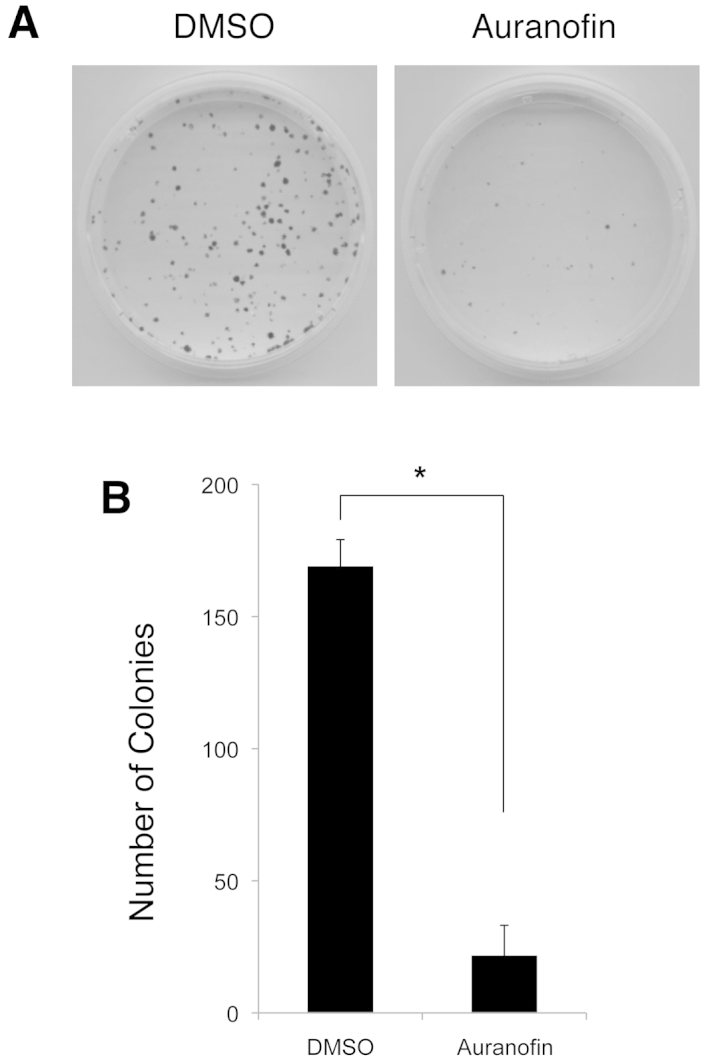
Auranofin suppresses the colony-forming ability of SKOV3 cells. The colony numbers were determined by the colony formation assay. (A) SKOV3 cells (500 cells/plate) were treated with auranofin (100 nM) or the control (DMSO) for 7 days and stained with crystal violet solution. The representative images of the assays are shown. (B) The numbers of colonies in the auranofin-treated plates were compared with those of the controltreated plates. The results are the mean ± SEM numbers of cell colonies of three replicates. ^*^P<0.001.

To examine the potential anticancer activity of auranofin against ovarian cancer cells, we treated human ovarian carcinoma SKOV3 cells with auranofin and measured the survival and/or growth rate of SKOV3 cells using the MTT, cell counting and colony formation assays. We showed that auranofin had an inhibitory effect on SKOV3 cell survival/growth in a dose- and time-dependent manner. After 72 h incubation, the dose-dependent assay data indicated that the IC_50_ value of auranofin on SKOV3 was ~150 nM (Fig. 2A), which is thought to be relatively low. The time-dependent assay results also demonstrated the anti-survival/ proliferation activity of auranofin (Fig. 2B), which was confirmed by cell counting assay using the same treatment condition of auranofin against SKOV3 cells (Fig. 3). After 120 h incubation, the cell number in SKOV3 cells treated with auranofin (100 nM) was significantly lower (~13 times) than that of the control (DMSO) treatment. Furthermore, the clonogenic assay results showed that auranofin treatment significantly suppressed the colony-forming ability of SKOV3 cells (Fig. 4). Taken together, these results support that auranofin displays a potent inhibitory effect on cell survival/proliferation of ovarian carcinoma SKOV3 cells.

### Auranofin induces cellular apoptosis in SKOV3 cells

**Figure 5:**
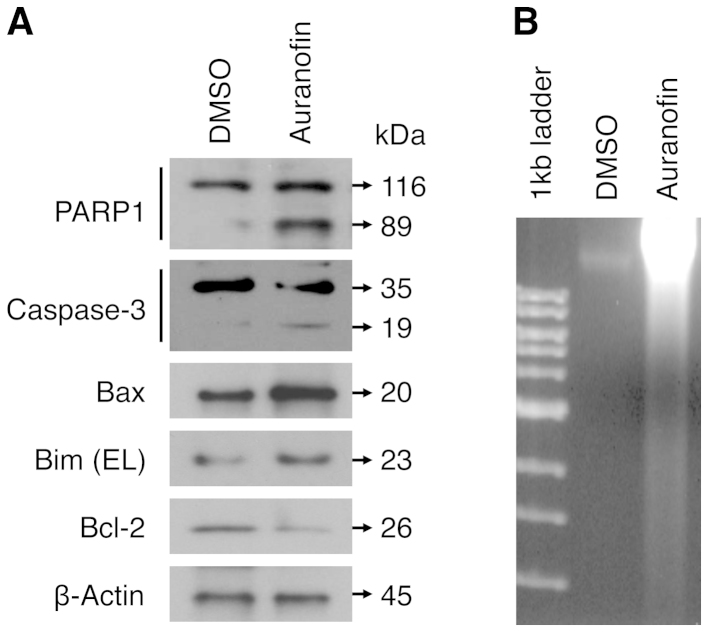
Auranofin induces cellular apoptosis in SKOV3 cells. (A) SKOV3 cells were treated with auranofin (100 nM) or control (DMSO) for 48 h. Total lysates of cells were analyzed by western blot analysis with specific antibodies against apoptosis-related proteins as indicated. β-actin represents the loading controls. (B) DNA samples extracted from SKOV3 cells, which were treated with auranofin or control as described above, and subjected to DNA fragmentation assay. Equal amounts of the extracted DNA (2 μg/lane) and size markers (1-kb ladder) were subjected to electrophoresis on 2% agarose gels, which were stained with ethidium bromide and photographed.

To examine the effect of auranofin on apoptosis in SKOV3 cells, we performed western blot analyses and DNA fragmentation assays. We found that the auranofin treatment (100 nM for 48 h) increased the cleavage of PARP1 and caspase-3. Auranofin also upregulated the expression of Bax and Bcl-2 interacting mediator of cell death (Bim) in SKOV3 cells as compared with the DMSO control treatment, whereas the auranofin treatment decreased the Bcl-2 expression level under the same condition (Fig. 5A). These results suggest that auranofin may exhibit its apoptotic effect through the caspase-3-mediated mechanism in SKOV3 cells, the upregulation of the mitochondrial proapoptotic Bax and Bim proteins and the downregulation of the anti-apoptotic Bcl2 protein expression. Also, when compared with the DMSO control, treatment of SKOV3 cells with auranofin (100 nM) for 48 h resulted in an increase in the amount of DNA fragmentation, a typical marker of apoptosis caused by the cleaved (active) caspase-3 (Fig. 5B). Collectively, our results show that auranofin treatment can induce cellular apoptosis in SKOV3 cells.

### Auranofin downregulates the expression of IKK-β and induces the nuclear translocation of FOXO3 protein in SKOV3 cells

**Figure 6:**
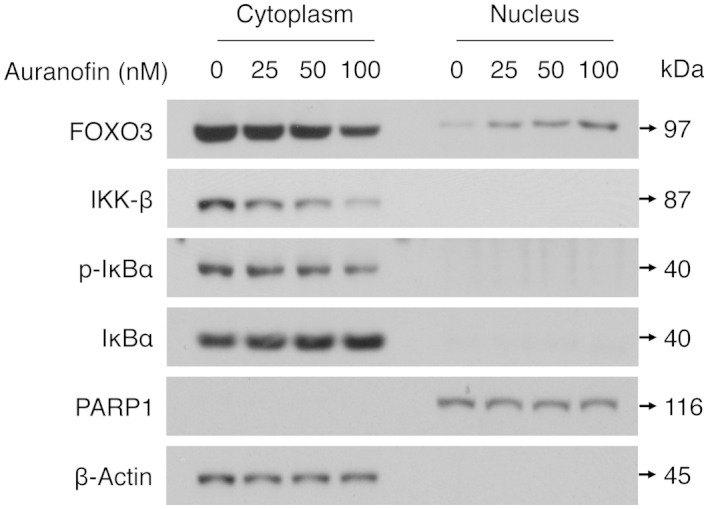
Auranofin downregulates IKK-β expression and promotes the nuclear translocation of FOXO3 protein in SKOV3 cells. SKOV3 cells were treated with auranofin (25, 50 and 100 nM) or control (0 nM) for 48 h and cytoplasmic and nuclear extracts that had been fractionated from cells were analyzed by western blot analysis with specific antibodies as indicated. β-actin and PARP1 represent the loading controls of cytoplasmic and nuclear extracts, respectively.

To elucidate the anticancer mechanism of auranofin treatment against SKOV3 cells, we carried out western blot analyses with cytoplasmic and nuclear extracts that had been fractionated from SKOV3 cells previously treated with auranofin. We showed that auranofin treatment (0, 25, 50 and 100 nM for 48 h) decreased the expression level of IKK-β protein in the cytoplasm in a dose-dependent manner. These data were confirmed by a parallel decrease of the phosphorylation level of IκBα, which is a well-known substrate of IKK-β, while the total amount of IκBα protein was not affected by auranofin treatment (Fig. 6). At the same time, the level of the cytoplasmic FOXO3 protein was decreased by auranofin treatment, while the level of the nuclear FOXO3 protein was significantly increased in a dose-dependent manner (Fig. 6). These results suggest that auranofin may display its anticancer effect through downregulation of IKK-β, which then triggers the translocation of the FOXO3 protein from the cytoplasm into the nucleus of ovarian cancer cells.

### Auranofin promotes apoptosis in SKOV3 cells in a FOXO3-dependent manner

**Figure 7:**
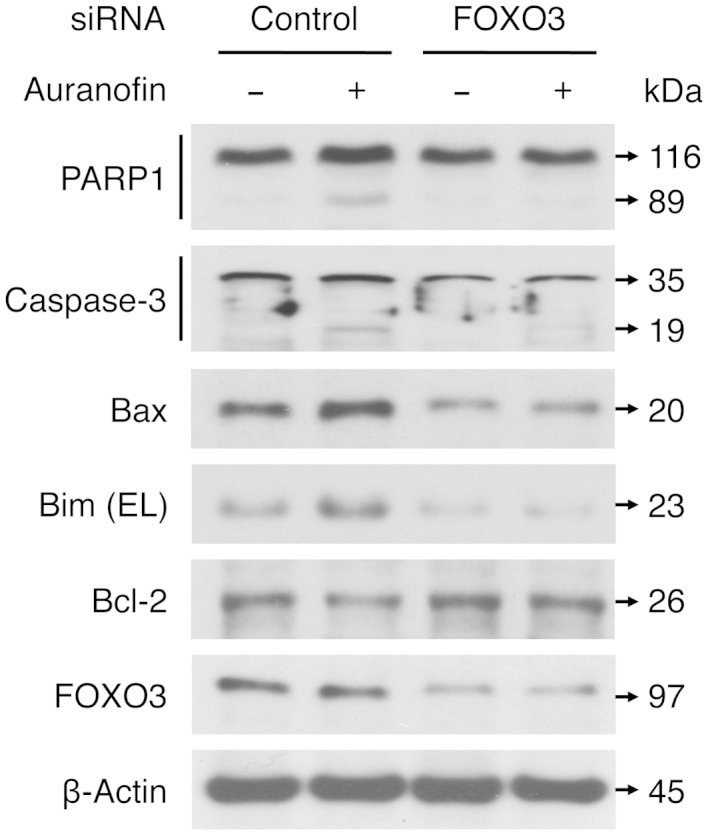
FOXO3 is essential for auranofin-mediated apoptosis in SKOV3 cells. SKOV3 cells were transfected with control-siRNA or FOXO3-siRNA for 24 h as described in Materials and methods. Subsequently, the transfected cells were treated with auranofin (100 nM) or control (DMSO) for 48 h and total lysates of cells were analyzed by western blot analysis with specific antibodies against apoptosis-related proteins as indicated. β-actin represents the loading controls.

To examine the role of FOXO3 in auranofin-induced apoptosis in SKOV3 cells, we silenced FOXO3 expression in SKOV3 cells and analyzed the protein status of PARP1, caspase-3, Bax, Bim, Bcl-2 and FOXO3 using western blot assays. Interestingly, knockdown of FOXO3 expression in SKOV3 cells significantly attenuated the caspase-3-mediated cleavage of PARP1 and caspase-3 proteins and reduced the expression levels of Bax and Bim, extra large (EL) isoform, when these cells were treated with auranofin (Fig. 7). In contrast, silencing FOXO3 decreases the repressive effect of auranofin on Bcl-2 expression. Collectively, these results suggest that FOXO3 may play an essential role in promoting the caspase-3-mediated apoptosis after auranofin treatment in SKOV3 cells.

## Discussion

**Figure 8:**
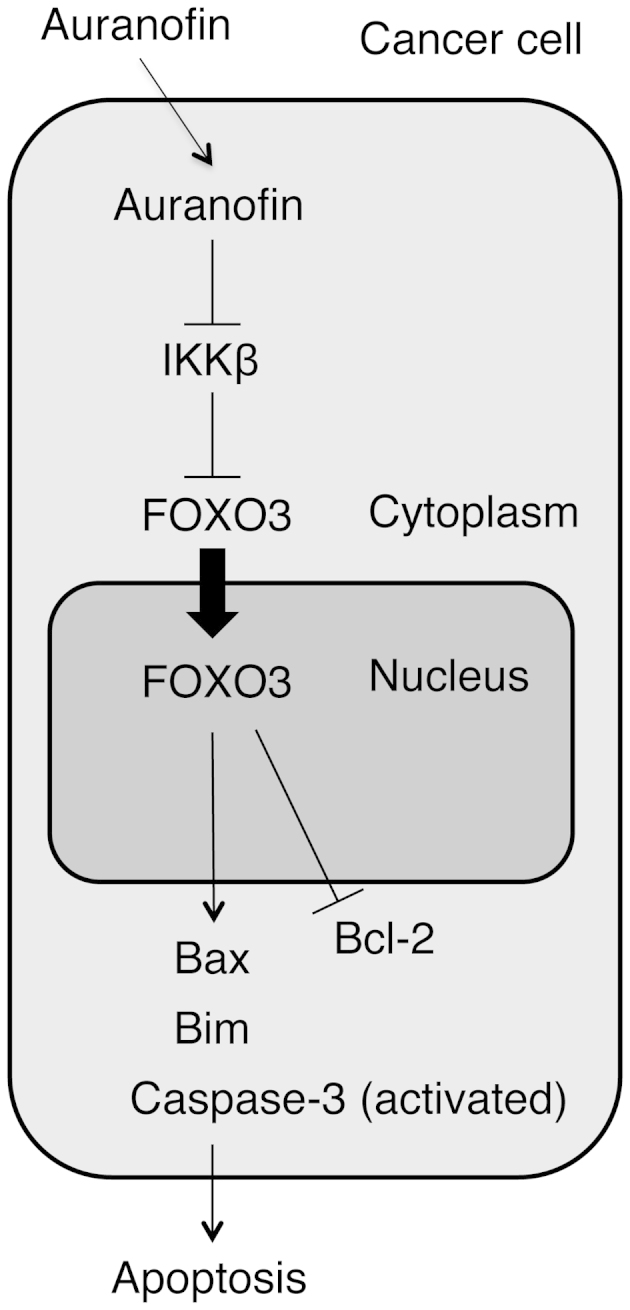
A diagram represents the model for the FOXO3-dependent anticancer function of auranofin. A schematic shows that auranofin inhibits IKK-β and leads to FOXO3 translocation from the cytoplasm into the nucleus to upregulate the expression of Bax and Bim pro-apoptotic proteins, as well as to downregulate of the expression of Bcl-2 anti-apoptotic protein. In addition, auranofin induces the activation of caspase-3 protein in a FOXO3-dependent manner. As a result of this FOXO3-mediated apoptotic pathway, auranofin promotes cancer cell apoptosis.

FOXO3 has received great attention as a potential prognostic biomarker for overall survival in patients with cancer because several clinical studies with primary tumor specimens have indicated that FOXO3′s nuclear exclusion or downregulation correlates significantly with poor prognosis and survival in breast and ovarian carcinomas (12,32–34). These findings also suggest that small molecules that can induce nuclear localization (activation) of FOXO3 in cancer cells may become promising antitumor chemotherapeutic drugs. The promotion of nuclear localization of the FOXO transcription factors by gold-containing compounds such as auranofin has not been reported and the roles of FOXO transcription factors in auranofin-mediated cellular apoptosis have not been determined. In this study, our results provide the first evidence that auranofin promotes the FOXO3 protein to translocate from the cytoplasm into the nucleus, where it upregulates the expression of the target genes Bax and Bim and downregulates the expression of Bcl-2 (an important gene regulating cell survival) in ovarian cancer cells. Because this phenomenon is discovered in a p53-null cell line SKOV3, it suggests that activation of FOXO3, Bax and Bim by auranofin may be through a mechanism independent of p53. It is known that the tumor suppressor p53 plays a key role in genotoxic stress responses including repair of DNA damage, cell cycle arrest and apoptosis, which are complicated and mostly through a p53-dependent pathway (38–40). Therefore, we propose a new mechanism by which FOXO3 induces apoptosis in ovarian cancer cells in response to auranofin treatment through upregulation of Bax and Bim and downregulation of Bcl-2 in a p53-independent manner (Fig. 8).

We found that auranofin treatment increased the cleavage of PARP1 and caspase-3 in SKOV3 cells, while the level of parental caspase-3 protein appear to be not significantly affected by auranofin treatment (Figs. 5A and 7). These results suggest that auranofin may exhibit caspase-3-mediated apoptotic effect through the activation of caspase-3 protein instead of through the upregulation of caspase-3 expression in SKOV3 cells. Interestingly, silencing the expression of FOXO3 in SKOV3 cells significantly reduced the caspase-3-mediated cleavage of PARP1 and caspase-3 proteins (Fig. 7). Currently, there is no literature to our knowledge that demonstrates FOXO3 is required for the caspase-3-mediated cleavage of PARP1 and caspase-3 proteins and for auranofin-induced apoptotic signaling in cancer cells. However, it remains largely unknown how FOXO3 regulates the activation of caspase-3 protein in cancer cells in response to auranofin treatment. It is of interest to further elucidate the molecular mechanisms governing the control of the FOXO3-mediated activation of caspase-3 protein in promoting the apoptotic signaling pathways in cancer cells.

As an anti-rheumatoid arthritis agent, auranofin has been shown to inhibit the activation of NF-κB by blocking IKK activity in the LPS-stimulated RAW 264.7 mouse macrophages (8). In this study, our results provide initial evidence of the anticancer effect of auranofin on human ovarian cancer cells, in which auranofin treatment downregulates the expression of IKK-β and reduces the level of phospho-IκBα (p-IκBα) (Fig. 6). Using the mutant forms of IKK-α and IKK-β subunits, Jeon *et al* suggest a possible inhibitory mechanism to explain how auranofin inhibited IKK activity *in vitro* (8). According to their report, a substitution of cystine-179 of IKK-β with alanine (mutant IKK-β-179A) made IKK-β resistant to inhibition by auranofin. However, a similar protective effect was not observed with IKK-α mutant. This result indicates that auranofin inhibited the two subunits of IKK in a different mode and that the inhibition of IKK activation induced by inflammatory signals in the auranofin-treated cells may be via its interaction with cystine-179 of IKK-β. It will be interesting to examine whether or not auranofin treatment exhibits an inhibitory effect on mutant IKK-β-179A activity in human cancer cells. Previously, our laboratory identified a novel mechanism by which cancer cells can impair the tumor suppressive function of FOXO3 protein by the oncogenic IKK-β-mediated phosphorylation of the serine-644 residue in FOXO3 (FOXO3-pS644) protein that leads to the translocation of FOXO3 from the nucleus into the cytoplasm in cancer cells (11,12). Subsequently, βTrCP1, an E3 Ub-ligase, interacts with the FOXO3-pS644 protein and induces the Ub-mediated degradation of FOXO3, resulting in the promotion of tumorigenesis and tumor growth *in vivo* (11). This IKK-β-mediated tumorigenic mechanism is consistent with and supports our current finding that auranofin treatment can downregulate the expression of IKK-β and promote FOXO3 nuclear localization to regulate the expression of its downstream target genes, resulting in the suppression of cell survival/growth and the promotion of cellular apoptosis in ovarian cancer cells.

From the standpoint of cancer therapy, the initial standard surgical management certainly plays an important and essential role in ovarian cancer treatment. Most patients will have appropriate surgical staging followed by optimal surgical cytoreduction, with the goal being to remove all gross disease before starting chemotherapy (2). There are a number of important issues to be resolved in the management of ovarian cancer that relates to surgery, including the role of surgical interval cytoreduction, as well as the role of second-look laparotomy (3). There are basically three initial chemotherapy options considered the standard of care at the present time for the treatment of ovarian cancer. The first is the use of carboplatin and paclitaxel. The second is a cisplatin and paclitaxel regimen. The third is the use of a carboplatin and docetaxel regimen. Unfortunately, in the second-line setting for the treatment of ovarian cancer, we have far fewer encouraging data based upon randomized controlled trials that give us definitive answers as to optimal management in the malignancy. A considerable number of patients harbor ovarian tumor refractory to these therapies and the majority of the initially responsive tumors become resistant to treatments (41). Thus, the development of novel targeted therapy that retains activity against chemotherapy-resistant ovarian cancer is an unmet medical need. Since auranofin is an FDA-approved small-molecule drug, it can be expedited for future clinical trials as a promising anticancer therapeutics and save time and money for the required pre-clinical investigation and toxicity testing for new compounds (42).

Finally, further investigation of the signaling mechanisms by which auranofin downregulates the expression of antiapoptotic proteins IKK-β and Bcl-2 that in turn contributes to tumor cell survival/growth may provide new cellular targets that can be exploited therapeutically. On the other hand, further elucidation of the molecular mechanisms underlying the FOXO3-mdiated pro-apoptotic signaling pathways induced by auranofin is necessary for understanding the molecular basis of auranofin anticancer activity and its application as a new anticancer therapy.
